# Patterns of heart rate variability and cardiac autonomic modulations in controlled and uncontrolled asthmatic patients

**DOI:** 10.1186/s12890-015-0118-8

**Published:** 2015-10-12

**Authors:** Mohamed Faisal Lutfi

**Affiliations:** Department of Physiology, Faculty of Medicine and Health Sciences, Al-Neelain University, Khartoum, Sudan

**Keywords:** Asthma control, Autonomic modulation, Heart rate variability

## Abstract

**Background:**

Previous heart rate variability (HRV) studies in asthmatic subjects (AS) demonstrate predominance of parasympathetic drive concomitant with low HRV, which is against the general belief that enhanced parasympathetic modulation improves HRV. The aim of this study was to compare patterns of HRV and cardiac autonomic modulations of AS to healthy control subjects (HS).

**Methods:**

Eighty AS and forty HS were enrolled in the study. Asthma control test and spirometry were used to discriminate uncontrolled (UA) from controlled (CA) asthmatic patients. Natural logarithmic (Ln) scale of total power (TP), very low frequency (VLF), low frequency (LF) and high frequency (HF) were used to evaluate HRV. Normalized low frequency (LF Norm) and high frequency (HF Norm) were used to determine sympathetic and parasympathetic autonomic modulations respectively.

**Results:**

CA patients achieved significantly higher LnTP, LnLF, LnHF and HF Norm but lower LF Norm and LnLF/HF compared with UA patients (*p* < 0.05). Although CA patients showed increased HRV and augmented vagal modulation compared with HS, these findings were no longer significant following adjustment for mean heart rates and anti-asthma treatment. All measured HRV parameters were not significantly different in UA patients compared with the HS (*p* > 0.05).

**Conclusions:**

CA is associated with enhanced parasympathetic modulations and higher HRV compared with UA. However, neither CA nor UA patients had different autonomic modulations and/or HRV compared with HS.

## Background

Assessment of heart rate variability (HRV) is one of the most popular methods used to evaluate autonomic modulations of the heart [1]. Recent research exploring deranged autonomic functions in certain diseases was largely based on HRV studies [2–4]. Bronchial asthma has received special attention by researchers investigating the impact of autonomic disturbances on the disease etiology [5–11]. Low HRV and predominance of parasympathetic modulations on the heart were demonstrated in some studies [5–8], but not others [9–11]. According to some previous reports, parasympathetic discharge seems to predominate in asthmatic patients irrespective of day-and-night cycle [12] or physical activity [5]. Conversely, some studies were unable to prove vagal hypertonia in patients with bronchial asthma [6]. Based on HRV derived indices, Gupta et al. failed to demonstrate parasympathetic predominance in asthmatic patients; however, their results suggested sympathetic withdrawal among the studied patients [6]. Garcia-Araújo and colleagues compared the autonomic modulation in 14 controlled asthmatic patients and 10 healthy volunteers at rest and during exercise. Their results suggested that controlled asthmatic patients had an increased sympathetic modulation and attenuated response to the postural changes [9]. In another study, HRV and cardiac autonomic modulations in difficult-to-control asthma patients were not different compared with healthy subjects [10].

In clinical practice, low HRV suggests increased susceptibility to cardiac arrhythmias secondary to autonomic imbalance [13, 14]. Enhanced parasympathetic drive is expected to increase HRV. Nonetheless, most studies in asthmatic patients that document shift of sympathovagal balance towards predominance of parasympathetic drive also demonstrate reduced HRV [5–8]. These contradictory findings are difficult to explain physiologically and may point to hidden confounders [15–18]. From a physiological standpoint, exploring variations in HRV in asthmatic patients should consider differences in heart rate [15, 16] and medical treatments [18, 19] among these patients. Altered heart rate is expected in asthmatic patients as a result of activation of the chemoreceptor reflex triggered by hypoxia/hypercapnia; sympathetic stimulation triggered by the stress of asthma attack; and beta-2 agonist therapy. Alternatively, heart rate is inversely proportional to beat-to-beat intervals, from which all HRV parameters are derived. Consequently, results of studies that do not adjust for asthma-associated changes in heart rates, and anti-asthma treatments (AAT), while comparing HRV and autonomic modulations between asthmatic and non-asthmatic subjects are questionable and deserve re-evaluation. Previous reports that failed to show predominance of vagal drive in asthmatic individuals may be biased by heterogeneous patterns of autonomic modulations among asthmatic patients [9–11]. Poor response of difficult-to-control asthma patients to anticholinergic drugs raises the question of whether enhanced vagal drive contributes to the pathogenesis of asthma in these patients [19, 20]. A negative answer is suggested by a recent study, which fails to demonstrate significant difference in the autonomic modulations between difficult-to-control asthma patients and healthy control subjects [10]. Further studies are required to verify if parasympathetic drive predominates in all asthmatic patients regardless of their disease control, AAT and asthma-associated changes in heart rates. The aim of this study was to compare patterns of HRV and cardiac autonomic modulations of asthmatic patients to healthy control subjects, taking into consideration possible influences of degree of asthma control, AAT and asthma-associated changes in heart rates on HRV.

## Methods

The study received ethical clearance from the ethics review committee at the Faculty of Medicine, University of Khartoum, Sudan. All volunteers provided informed written consent before being involved in the study.

Eighty (41 males and 39 females) known asthmatic subjects (AS) with no past medical history of other chronic pulmonary diseases were matched for age, gender, weight and height with a control group of 40 (22 males and 18 females) apparently healthy subjects (HS). All studied individuals were young adults, and had no past medical history of smoking, diabetes mellitus, hypertension, heart disease or other diseases that may affect cardiac autonomic modulations. The test group was recruited from respiratory referral clinics of the Khartoum teaching hospitals, Sudan. The control subjects were students and doctors of the Faculty of Medicine and Health Sciences, Al-Neelain University, Khartoum, Sudan.

Following evaluation of past medical history, including intensity of anti-asthma treatment offered to the patients, all volunteers underwent clinical examination and investigations during the period from 09.00 AM–12.00 PM  to avoid confounding effects of circadian rhythm on asthma severity, spirometry and HRV. Using asthma control test (ACT), a score of 15 or less was used to discriminate uncontrolled (UA) from controlled asthmatic (CA) patients [21, 22]. For further verification of ACT, ventilatory function was evaluated in each class using spirometry. For the purpose of adjustment for anti-asthma medications, patients were further categorized into: off-treatment patients, patients on beta-2 agonists only and patients on combined beta-2 agonists and steroids. A GIMA scale (GIMA S.p.A, Milan, Italy) was used to measure weight and height. Body mass index (BMI) was determined by the formula: BMI (kg/m^2^) = weight/height squared. Spirometry and HRV were performed according to the standard methods using Allflow Spirometer (Version 5.18, Clement Clarke International Limited, Harlow, UK) and Biocom 3000 ECG recorder (Heart Rhythm Scanner, Version 2.0, Biocom Technologies, Poulsbo, WA, U.S.A) respectively [15, 23].

HRV parameters were derived from 5-min electrocardiogram (ECG) recordings in the supine position after ensuring clean ECG signals, absence of movement artifacts and comfortable breathing. Frequency domain analysis was used to determine HRV and cardiac autonomic modulations in the different groups. Natural logarithm (Ln) of total power (LnTP), very low frequency (LnVLF), low frequency (LnLF) and high frequency (LnHF) were used to evaluate HRV. Alternatively normalized low frequency (LF Norm) and high frequency (HF Norm) were used to determine sympathetic and parasympathetic autonomic modulations respectively [24]. The Biocom 3000 ECG recorder also calculated the mean heart rate (MHR) at the time of ECG recording.

### Statistical analysis

Statistical analysis was done using SPSS for windows (Version 16; Chicago, IL, USA). Variables were described by means, median, standard deviations (SD), 25 quartile (Q1) and 75 quartile (Q3) based on their distribution curves. Using appropriate statistical tests, comparable distributions of gender, ages and BMI among control and test groups were established. Concordance of spirometric measurements with ACT groups was ensured to approve proper grading of asthma severity by ACT. Means (SD) and median (Q1-Q3) of frequency domain HRV parameters and spirometric measurements were compared in the studied groups using one-way ANOVA (Student’s *T* test for comparison between two groups) and Kruskal–Wallis (Mann–Whitney *U* test for comparison between two groups) tests, respectively. Using a general linear model MHR, and AAT for each subject were introduced as covariates while comparing each HRV parameter in the different studied groups. A value of *P* < 0.05 was considered significant.

## Results

**Fig. 1:**
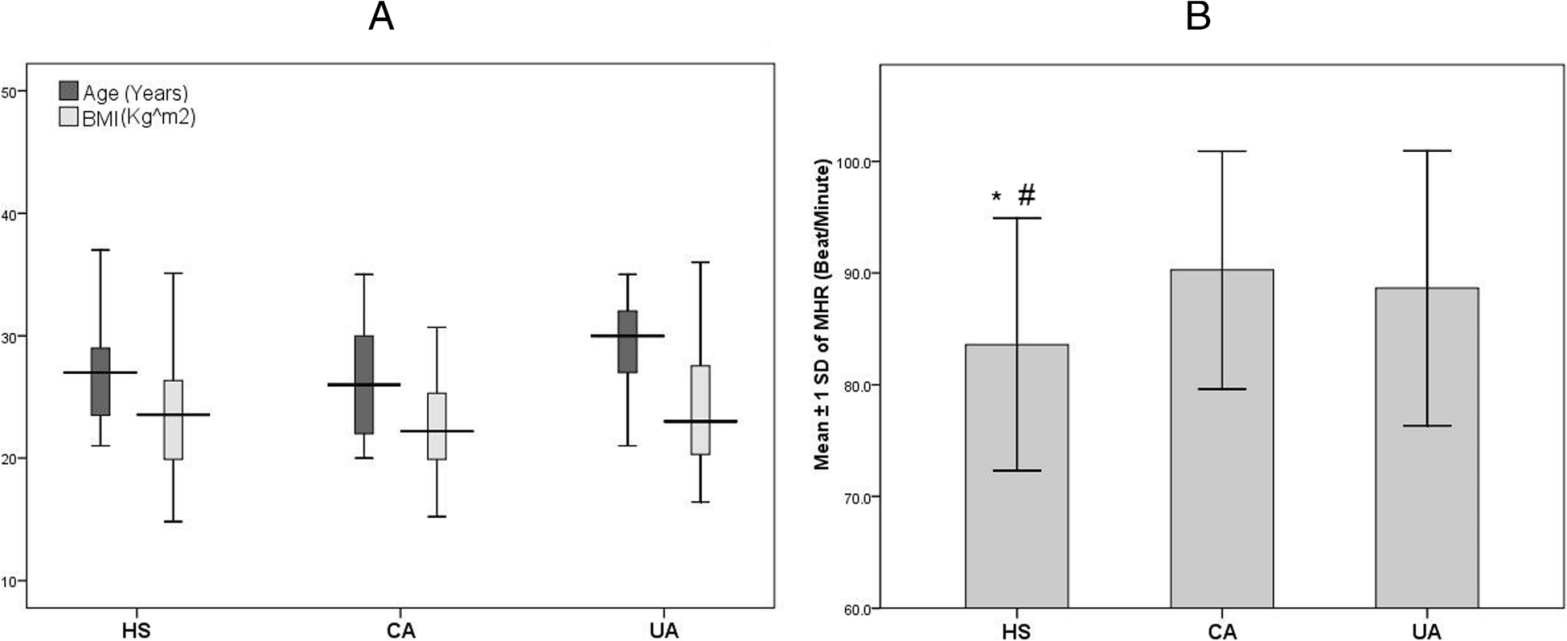
Distribution of age, BMI (**a**) and MHR (**b**) among studied groups. * Significant compared with CA, # significant compared with UA

Of the AS (*n* = 80), 37 [46.3; 95 % confidence interval (CI): 35.8, 57.1 %] had CA and 43 (53.7; 95 % CI: 42.9, 64.3 %) had UA. The distribution of gender (*chi*^*2*^ = 0.15, *p* = 0.698), ages, and BMI were comparable in AS and HS. In contrast, MHR was significantly lower in HS [83.6 (11.3) beats/min] compared with CA [90.3 (10.6) beats/min, *p* = 0.012] and UA [88.6 (12.3) beats/min, *p* = 0.048] (Fig. 1). As shown in Table 1, all measured spirometric indices were significantly increased in HS compared with CA and UA. Except for peak expiratory flow rate, the same spirometric measurements were significantly increased in CA compared with UA.

**Table 1:** ACT score and spirometric measurements among studied groups

	HS	CA	UA	*P*
	*N* = 40	*N* = 37	*N* = 43	
	Mean (SD)	Mean (SD)	Mean (SD)	
	Median (Q1 –Q3)	Median (Q1 –Q3)	Median (Q1 –Q3)	
ACT score		19.0 (17.0 – 21.0)	13.0 (10.0–13.0)	<0.001*
FEV1%	90.5 (5.5)	78.5 (14.6)	68.0 (13.6)	HS vs. CA < 0.001*
				HS vs. UA < 0.001*
				CA vs.UA < 0.001*
PEFR (L/sec)	7.4 (1.6)	5.3 (1.9)	4.5 (1.9)	HS vs. CA < 0.001*
				HS vs. UA < 0.001*
				CA vs.UA = 0.054
FEF25% (L/sec)	6.8 (1.5)	4.7 (2.0)	3.5 (1.8)	HS vs. CA < 0.001*
				HS vs. UA < 0.001*
				CA vs.UA = 0.006*
FEF50% (L/sec)	4.9 (3.7–6.1)	2.8 (2.0 – 4.4.0)	1.7 (1.0–2.6)	HS vs. CA < 0.001*
				HS vs. UA < 0.001*
				CA vs.UA = 0.004*
FEF75% (L/sec)	2.3 (1.8–2.5)	1.2 (0.7 – 1.8)	0.7 (0.4–1.3)	HS vs. CA < 0.001*
				HS vs. UA < 0.001*
				CA vs.UA = 0.005*

*ACT* Asthma control test, *CA* Controlled asthmatic, *FEF* Forced expiratory flow, *FEV1* Forced expiratory volume in the first second, *HS* Healthy subjects, *PEFR* Peak expiratory flow rate, *Q1* 25 quartile, *Q3* 75 quartile, *SD* Standard deviation, *UA* Uncontrolled asthma, * Statistically significant.

CA patients achieved significantly higher LnTP, LnLF, LnHF and HF Norm [7.40 (1.27) ms^2^/Hz, 6.23 (1.30) ms^2^/Hz, 6.41 (1.65) ms^2^/Hz and 54.4 (16.4) normalized unit (nu) respectively] compared with the HS [6.68 (1.31) ms, *p* = 0.023; 5.50 (1.43), *p* = 0.026; 5.32 (1.76) ms, *p* = 0.008 and 45.9 (17.3) nu, *p* = 0.026 respectively] as well as UA patients [median 6.64 (1.49) ms, *p* = 0.015; 5.53 (1.47) ms, *p* = 0.030; 5.31 (1.84) ms, *p* = 0.006 and 45.8 (17.6) nu, *p* = 0.026 respectively] (Table 2).

**Table 2:** Comparison of HRV parameters between groups

	HS	CA	UA	*P* values		
	*N* = 40	*N* = 37	*N* = 43	Non-Adjusted	Adjusted for MHR	Adjusted for MHR and AAT
	Mean (SD)	Mean (SD)	Mean (SD)			
LnTP (ms2/Hz)	6.68 (1.31)	7.40 (1.27)	6.64 (1.49)	HS vs. CA = 0.023*	HS vs. CA = 0.037*	HS vs. CA = 0.673
				HS vs. UA = 0.885	HS vs. UA = 0.857	HS vs. UA = 0.314
				CA vs.UA = 0.015*	CA vs.UA = 0.019*	CA vs.UA = 0.020*
LnVLF (ms2/Hz)	5.47 (1.12)	5.87 (1.02)	5.36 (1.47)	HS vs. CA = 0.157	HS vs. CA = 0.163	HS vs. CA = 0.821
				HS vs. UA = 0.673	HS vs. UA = 0.950	HS vs. UA = 0.481
				CA vs.UA = 0.065	CA vs.UA = 0.076	CA vs.UA = 0.080
LnLF (ms2/Hz)	5.50 (1.43)	6.23 (1.30)	5.53 (1.47)	HS vs. CA = 0.026*	HS vs. CA = 0.042*	HS vs. CA = 0.658
				HS vs. UA = 0.911	HS vs. UA = 0.668	HS vs. UA = 0.141
				CA vs.UA = 0.030*	CA vs.UA = 0.033	CA vs.UA = 0.031*
LnHF (ms2/Hz)	5.32 (1.76)	6.41 (1.65)	5.31 (1.84)	HS vs. CA = 0.008*	HS vs. CA = 0.016*	HS vs. CA = 0.490
				HS vs. UA = 0.979	HS vs. UA = 0.761	HS vs. UA = 0.346
				CA vs.UA = 0.006*	CA vs.UA = 0.007*	CA vs.UA = 0.008*
LF Norm (nu)	54.1 (17.3)	45.6 (16.4)	54.2 (17.6)	HS vs. CA =0.021*	HS vs. CA =0.053	HS vs. CA =0.406
				HS vs. UA =0.748	HS vs. UA =0.896	HS vs. UA =0.716
				CA vs.UA =0.041*	CA vs.UA =0.025	CA vs.UA =0.030*
HF Norm (nu)	45.9 (17.3)	54.4 (16.4)	45.8 (17.6)	HS vs. CA =0.031*	HS vs. CA =0.053	HS vs. CA =0.406
				HS vs. UA =0.983	HS vs. UA =0.896	HS vs. UA =0.716
				CA vs.UA =0.026*	CA vs.UA =0.025*	CA vs.UA =0.030*
LnLF/HF	0.19 (0.76)	−0.19 (0.75)	0.22 (0.86)	HS vs. CA = 0.040*	HS vs. CA = 0.054	HS vs. CA = 0.438
				HS vs. UA = 0.838	HS vs. UA = 0.983	HS vs. UA = 0.558
				CA vs.UA = 0.022*	CA vs.UA = 0.025*	CA vs.UA = 0.029*

*AAT* Anti-asthma treatments, *CA* Controlled asthmatic, *HF* High frequency, *HF Norm* normalized high frequency, *HRV* Heart rate variability, *HS* Healthy subjects, *LF* Low frequency, *LF Norm* normalized low frequency, *Ln* Natural logarithm, *MHR* Mean heart rate, *SD* Standard deviation, *TP* Total power, *UA* Uncontrolled asthma, *VLF* Very low frequency, * Statistically significant.

LF Norm and LnLF/HF were significantly lower in CA patients [45.6 (16.4) nu and −0.19 (0.75) respectively] compared with the HS [54.1 (17.3) nu, *p* = 0.021 and 0.19 (0.76), *p* = 0.040 respectively] as well as UA patients [54.2 (17.6) nu, *p* = 0.041 and 0.22 (0.86), *p* = 0.022 respectively]. All measured frequency domain HRV parameters were not significantly different in UA patients compared with HS (Table 2).

All HRV differences between the groups were still significant following adjustment for MHR at the time of HRV recording, except for LF Norm, HF Norm and LnLF/HF, which became comparable in HS and CA patients (Table 2). Adjustment for MHR and AAT nullified all significant variations between HS and CA but all other differences between CA and UA patients remained statistically significant (Table 2).

## Discussion

It is evident from the present results that global HRV and autonomic modulations of CA patients are significantly different from UA patients irrespective of the possible influences of MHR and AAT on HRV. Based on absolute and normalized HRV parameters, CA patients had higher global HRV and augmented parasympathetic autonomic modulations compared with UA patients. Comparing HS to AS, it is clear that global HRV and autonomic modulations of UA patients were comparable to the HS group regardless of influences of MHR and AAT on HRV. Although CA patients showed higher HRV and augmented vagal modulation compared with HS, these findings were no longer significant following adjustment for MHR and AAT.

Several previous reports confirm predominance of the parasympathetic nervous system on cardiac autonomic modulations of asthmatic patients [5, 6, 25–27]. Gupta et al. compared findings of spectral analysis HRV of 30 asthmatic patients aged 20–30 years to a similar number of healthy volunteers. Their results confirmed lower LF Norm in asthmatic patients compared with the healthy volunteers but failed to demonstrate significant difference in HF Norm among the studied groups [6]. These findings were further supported by the work of Gomes et al. who failed to demonstrate significant decrease in HF power following shuttle walk tests offered to asthmatic children. From an autonomic standpoint, the Gomes et al. findings suggested predominance of parasympathetic cardiac modulations even during exercise, when sympathetic effects were expected to dominate [5]. In another study, circadian rhythm of parasympathetic nervous function disappeared in 9 out of the 80 asthmatic children examined by Kazuma et al. but was observed in all healthy children [12]. Absence of parasympathetic circadian rhythm in some asthmatic children is probably due to persistent high parasympathetic discharge. Interestingly, enhanced parasympathetic cardiac modulations were proven in patients who are at higher risk to develop asthma, e.g., those with allergic rhinitis [28] and atopic dermatitis [29].

It is worth mentioning that the results of this study stand midway between the findings of previous researchers exploring autonomic modulations in asthmatic patients. This is because the present results suggested augmented parasympathetic and depressed sympathetic autonomic modulations in CA compared with UA patients but fail to demonstrate similar differences in autonomic trends when the AS group was compared with HS. To our knowledge, the present study is the first report to state clearly different patterns of cardiac autonomic modulations among controlled and uncontrolled asthmatic patients, although some previous reports gave cues that may support the present hypothesis [10, 11]. Cabiddu et al. studied HRV in eight patients suffering from difficult-to-control asthma during different stages of sleep [10]. In healthy subjects in this study, HRV showed predominant parasympathetic drive during non-rapid eye movement sleep and an increased sympathetic activity during rapid eye movement sleep. Spectral analysis of HRV in difficult-to-control asthma patients showed trends in the main spectral indices comparable to the control subjects [10].

According to the present results, CA patients have higher global HRV compared with UA subjects but the HRV of either asthma group was comparable to HS. Conversely, previous reports repeatedly demonstrated reduced HRV among asthmatic patients compared with healthy individuals [7, 9, 12, 30]. The incongruity of our results with previous reports might be explained by the unique design of our study which considered adjustment for MHR and AAT while comparing AS to HS. Alternatively, higher global HRV of CA compared with UA subjects could be explained by the findings of a recent study designed by Domnik et al. to assess the impact of allergic sensitization to ovalbumin on HRV in a murine model [11]. The researchers were able to demonstrate that sensitized mice had decreased HRV prior to ovalbumin challenge, increased HRV during antigen challenge and finally decreased HRV subsequent to the challenge. Based on these results, HRV is likely to increase in controlled asthmatic patients who usually suffer from acute intermittent exacerbations but not uncontrolled patients, who usually suffer from persistent attacks. This conclusion may explain why TP, LF and HF powers were higher in CA patients examined in this study compared with the UA subjects. Another salient point in this context is that enhanced parasympathetic cardiac autonomic modulations are known to improve, rather than to weaken, HRV [13, 14]. It appears logical that augmented parasympathetic cardiac autonomic modulations in CA asthmatic patients will improve global HRV, as proved by our results, and not depress HRV, as demonstrated by previous studies.

The difference in the patterns of autonomic modulations between CA and UA patients suggests synonymous variation in the pathophysiological processes involved in each group. In CA patients, enhanced parasympathetic and attenuated sympathetic activity will aggravate bronchoconstriction of the respiratory tree and consequently worsen airways narrowing. According to the present results, parasympathetic autonomic discharge in UA patients is less than CA and comparable to HS suggesting that other mechanisms are involved in inducing airways obstruction in UA patients. Previous reports suggested that UA patients are difficult to treat because they express unique pathological myofilaments in bronchiolar smooth muscles [31, 32] beside other airway changes [33, 34]. In addition, the variations in the pattern of autonomic modulations among asthmatic patients explain differences in the response of asthma to conventional drugs that act on the autonomic nervous system, e.g. UA patients may respond badly to parasympatholytic drugs [19, 20].

Although it is widely accepted that the HF power is a reliable indicator of parasympathetic modulations, the validity of the LF power as a measure of sympathetic modulations is a more contentious issue [35]. There is growing evidence that the absolute values of LF are determined by baroreflexes and therefore may efficiently reflect parasympathetic tone [36]. The interpretation of the present results was partly based on the normalized LF and HF which, in contrast to their absolute values, are inversely proportional and therefore may better reflect sympathetic and parasympathetic modulations [24]. Another limitation of this study is that respiratory rates were not acknowledged in different study groups while comparing their HRV. HRV is modulated by breathing and evaluation of the rate of respiration in future studies could offer better understanding for autonomic balance in asthmatic patients.

## Conclusions

The present results confirmed that degree of asthma control influences pattern of autonomic modulations/HRV among AS. Enhanced parasympathetic modulations offered higher HRV to CA compared with UA patients who suffered from attenuated vagal tone. However, neither CA nor UA patients were shown to have different autonomic modulations and/or HRV compared with HS. The comparable autonomic modulations/HRV among AS and HS, although different to several previous reports, were based on a well-designed comparison that considered possible influences of MHR and AAT on HRV.

## Abbreviations

AAT: Anti-asthma treatments
ACT: Asthma control test
AS: Asthmatic subjects
BMI: Body mass index
CA: Controlled asthmatic
HF: High frequency
HF Norm: Normalized high frequency
HRV: Heart rate variability
HS: Healthy subjects
LF: Low frequency
LF Norm: Normalized low frequency
Ln: Natural logarithm
MHR: Mean heart rate
Q1: 25 quartile
Q3: 75 quartile
TP: Total power
UA: Uncontrolled asthma
VLF: Very low frequency
